# Long Term Outcome After Open Abdomen Treatment: Function and Quality of Life

**DOI:** 10.3389/fsurg.2021.590245

**Published:** 2021-03-29

**Authors:** Alexis Theodorou, Agnes Jedig, Steffen Manekeller, Arnulf Willms, Dimitrios Pantelis, Hanno Matthaei, Nico Schäfer, Jörg C. Kalff, Martin W. von Websky

**Affiliations:** ^1^Department of General, Visceral, Thoracic and Vascular Surgery, University Hospital Bonn, Bonn, Germany; ^2^Department of General-, Visceral- and Thoracic Surgery, Bundeswehr Central Hospital, Koblenz, Germany

**Keywords:** open abdomen treatment, abdominal compartment syndrome, long term outcome, planned ventral hernia, peritonitis, SCAR, enteroatmospheric fistula

## Abstract

**Background:** Open abdomen treatment (OAT) is widely accepted to manage severe abdominal conditions such as peritonitis and abdominal compartment syndrome but can be associated with high morbidity and mortality. The main risks in OAT are (1) entero-atmospheric fistula (EAF), (2) failure of primary fascial closure, and (3) incisional hernias. In this study, we assessed the long-term functional outcome after OAT to understand which factors impacted most on quality of life (QoL)/daily living activities and the natural course after OAT.

**Materials and Methods:** After a retrospective analysis of 165 consecutive OAT patients over a period of 10 years (2002–2012) with over 65 clinical parameters that had been performed at our center (1), we initiated a prospective structured follow-up approach. All survivors were invited for a clinical follow-up. Forty complete datasets including clinical and social follow-up with SF-36 scores were available for full analysis.

**Results:** The patients were dominantly male (75%) with a median age of 52 years. Primary fascial closure (PC) was achieved in 9/40 (23%), while in 77% a planned ventral hernia (PVH) approach was followed. A total of 3/4 of the PVH patients underwent a secondary-stage abdominal wall reconstruction (SSR), but 2/3 of these reconstructed patients developed recurrent hernias. Fifty-five percent of the patients with PC developed an incisional hernia, while 20% of all patients developed significant scarring (Vancouver Scar Score >8). Scar pain was described by 15% of the patients as “moderate” [Visual Analog Scale (VAS) 4–6] and by 10% as “severe” (VAS > 7). While hernia presence, PC or PVH, and scarring showed no impact on QoL, male sex and especially EAF formation significantly reduced QoL.

**Discussion:** Despite many advantages, OAT was associated with relevant mortality and morbidity, especially in the early era before the implementation of a structured concept at our center. Follow-up revealed that hernia incidence after OAT and secondary reconstruction were high and that 25% of patients qualifying for a secondary reconstruction either did not want surgery or were unfit. Sex and EAF formation impacted significantly on QoL, which was lower than in the general population. With regard to hernia incidence, new strategies such as prophylactic mesh implantation upon fascial closure should be discussed analogous to other major abdominal procedures.

## Introduction

In the recent years, open abdomen treatment (OAT) has become a widely accepted treatment strategy for severe abdominal conditions such as peritonitis and abdominal compartment syndrome (1). However, OAT can be associated with inherent high morbidity and mortality (2). Atema et al. reported in a recent review of OAT in non-trauma patients an overall mortality rate of 30% (3). The main procedure-inherent risks in patients undergoing OAT are (1) the development of an entero-atmospheric fistula (EAF), (2) failure of primary fascial closure (PC) resulting in a planned ventral hernia (PVH), and (3) high rates of incisional hernias after PC. Recent studies have demonstrated that a structured approach including (a) the use of a visceral protection layer, (b) mesh-mediated fascial traction, and (c) negative pressure wound treatment reduces the above-mentioned complications significantly (4).

The rate of incisional hernia development after primary fascial closure in OAT may be higher than in usual laparotomy which has an incidence of 5–20% in the general patient population (5). In OAT, incisional hernia incidence after PC was reported to be as high as 35–65% (6). Incisional hernia rates of this proportion are also known for other high-risk situations such as abdominal aortic aneurysm repair or obese patients (7). While in the early era of OAT a planned ventral hernia was often accepted as unavoidable, recent evidence shows that achieving PC as soon as possible is associated with reduced complications (2). For example, The World Society of Emergency Surgery suggests early fascial closure as the key strategy for the management of open abdomen with a grade 1B recommendation (8). The recent literature also suggests that early closure should be achieved within 10 days (4, 9). Thus, while hospital discharge with PVH after OAT becomes less frequent, the incidence of an incisional hernia after open abdomen treatment is high (6). The presence of an incisional hernia is associated with a higher rate of readmissions and subsequent operations (10). Furthermore, patients with incisional hernias experience a lower health-related quality of life (QoL) on physical components and a worse body image (11). It is unclear what can be done to prevent incisional hernias after OAT, and this aspect will receive more attention as high rates of delayed primary closure in OAT become more and more feasible.

As mentioned above, the second major problem after OAT is presented by the formation of entero-atmospheric fistulas (incidence of 7 to 19%), which is associated with high morbidity and mortality (2, 12). A prospective analysis of the International Register of Open Abdomen from Coccolini et al. has shown that EAF formation is—among other factors—potentially influenced by the duration of OAT, the patients' characteristics (such as malignancy or inflammatory bowel disease), and the timing of restarting enteral nutrition. Despite the caution regarding an untoward effect of negative pressure on hollow viscera, Coccolini et al. showed no existing link between negative pressure treatment and EAF development (12). A study by our working group showed that the combined use of a visceral protection layer and negative pressure wound treatment effectively reduced the formation of EAF formation in OAT patients with peritonitis (4).

Historically, the traditional method to close the OAT-induced fascial defect was to neglect midline closure, let a ventral hernia develop, and then repair this hernia in a secondary-stage abdominal wall reconstruction 6 to 12 months later (PVH approach). This technique was often combined with a temporary abdominal closure using an absorbable or non-absorbable mesh and negative pressure wound therapy. Hereby the laparotomy is allowed to granulate, followed in some cases by split-thickness skin grafting (13, 14). Logically, this results in excessive scarring. Multiple studies in burn damage survivors have shown that abnormal scarring can be associated with reduced QoL (15). For OAT, however, compiled data on esthetic and functional outcomes including scarring by using an objective score (Vancouver Scar Scale, VSS) were not available.

In this study, we assessed the long term clinical, functional and QoL outcome in OAT patients of the early era at our institution to understand which factors (PVH *vs*. PC, EAF formation, scarring, recurrent incisional hernia) impacted most on QoL and the natural course after OAT.

## Methods

The primary study was conceived in 2012, after consultation with the local ethics committee. As a first step, data were systematically gathered from all medical records of 174 patients that underwent OAT in our hospital (University Hospital of Bonn) over a period of 10 years (2002–2012) for different indications (Supplementary Figure 1). After quality control, 165 patient records were available for full analysis, and more than 65 clinical variables were extracted from the records. The overall results of the retrospective analysis are published elsewhere (16). Over time, the patients had been treated with different approaches for OAT according to era. The earliest cohort of the patients was often treated using a PVH approach using an absorbable polyglactin (Vicryl) mesh as a temporary abdominal closure, with a planned secondary-stage abdominal wall reconstruction at the earliest after 6 months. The most recent cohort of patients was treated using a standardized algorithm (“Koblenz algorithm”) that uses a combination of mesh-mediated fascial traction, visceral protection, and vacuum-assisted wound closure (17). To address long-term outcome, we initiated a structured follow-up approach with telephone and written contact and invited all 95 survivors of the historic cohort for a clinical follow-up. The patients who were willing to participate after informed consent received the German 36-Item Short Form Health Survey (SF-36) questionnaire to assess QoL. The widely accepted SF-36 relies on patient self-reporting and consists of eight scaled scores, which are the weighted sums of the questions in their section. Each scale is directly transformed into a 0–100 scale on the assumption that each question carries an equal weight: the lower the score the more disability and, *vice versa*, the higher the score the less disability is displayed.

A total of 53 patients were not available or did not respond to our contact attempts. We performed a clinical follow-up examination in 42 patients of that cohort but had to exclude two patients due to incomplete SF-36 data. Thus, 40 complete datasets including clinical follow-up were available for analysis (Supplementary Figure 1). The median follow-up time of these 40 patients in this follow-up was 4.4 years after the index operation and OAT. In our clinical follow-up examination, QoL was assessed by SF-36 as mentioned, the presence of a clinically relevant incisional hernia was recorded, and an objective scar assessment using the VSS was performed. The modified Vancouver Scar Scale provides a standardized assessment of scarring. It scores the scar on four parameters: pigmentation, vascularity, pliability, and height (18). In addition, we used a verbal numerical rating scale as an assessment method of scar-related pain and itching in those patients.

In this study, we thus report the outcome of 40 long-term survivors of OAT concerning functional, esthetic, and QoL outcome, including data on primary fascial closure and method, presence of EAF, hernia presence, scar condition, and QoL as assessed with the SF-36 questionnaire.

## Statistics

Descriptive and inferential statistics were used in data analysis using SPSS Statistics Version 24 (IBM, Armonk, New York, USA). Intergroup differences were calculated for the SF-36 score using Students' *t*-test followed by Bonferroni correction. Clinical parameters were analyzed for possible correlations using Pearson‘s correlation coefficient. *P*-values were two-sided, and statistical significance was set at 0.05.

## Results

### Epidemiology

The epidemiologic and clinical data of the 40 patients participating in the follow-up study are presented in Table 1. Median patient age was 52 years, and sex was predominantly male.

**Table 1:** Epidemiologic data, comorbidities and potential influencing factors of postoperative outcome.

***N* total = 40**	***n*, (%)**
Sex:	
m	30 (75)
f	10 (25)
Malignancy at time of index procedure	5
ASA (19) at time of index procedure:	
I	0 (0)
II	7 (18)
III	23 (58)
IV	9 (23)
V	1 (1)
Index Procedure:	
Colorectal	11 (28)
Pancreas	12 (30)
Small bowel	3 (7)
HPB	6 (15)
Other	8 (20)
Indication for OAT:	
Peritonitis/anastomotic leakage	22 (55)
Hemorrhage	3 (7)
Pancreatitis	7 (18)
Abdominal compartment syndrome	3 (7)
Other	5 (13)
Obesity (BMI >30 kg/m^2^)	10 (25)
Cardiovascular disease	14 (35)
Immunosuppression	3 (7)
Renal failure	5 (13)
Prior malignancy	9 (23)
Lung disease	2 (5)
Diabetes mellitus	7 (18)
Prior abdominal surgery	16 (40)

*Peritonitis was the most common indication for open abdomen treatment*.

### Clinical Course

The median hospital stay was 71 days, and the median duration of OAT (from index operation until the closure of the abdominal wall) was 13 days, and this was achieved with a median of 6.5 procedures (scheduled reoperations). The survival rate of the entire historic patient cohort was 57%. Seven (18%) of the patients developed an entero-atmospheric fistula at some point along the duration of OAT. A vacuum-assisted wound closure method was used in 25 cases (63%).

### Fascial Closure and Hernia Development

**Figure 1:**
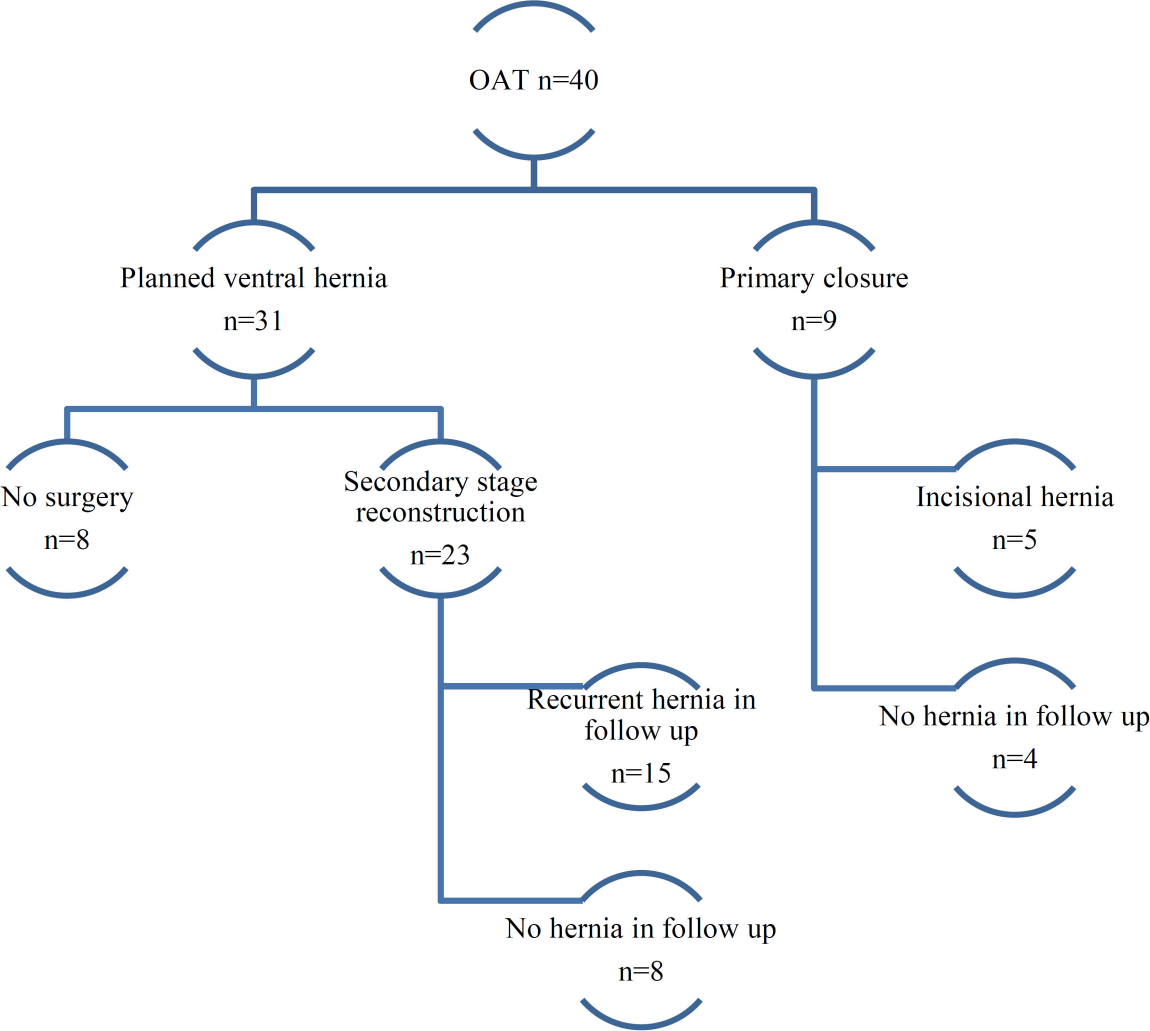
Abdominal closure and hernia incidence after open abdomen treatment (OAT). The flowchart shows the results of OAT concerning primary closure, planned ventral hernia, and recurrent hernia incidence.

**Figure 2:**
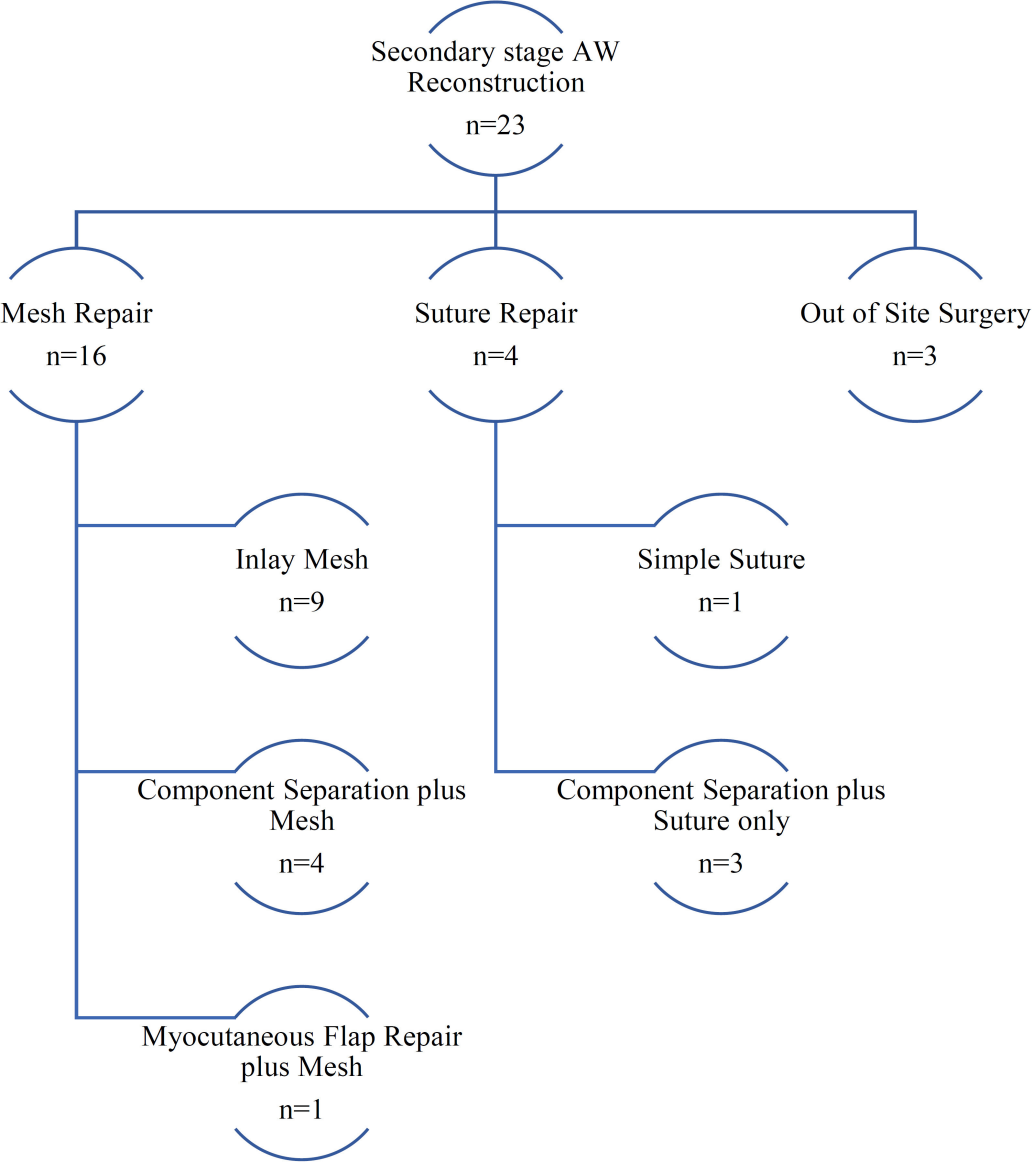
Secondary-stage abdominal wall reconstruction techniques. The flowchart shows a synopsis of techniques used for secondary-stage abdominal wall reconstruction at our center.

Primary closure was achieved in nine cases (22%), and a planned ventral hernia approach had to be employed in 31 patients (Figure 1). Twenty-three of the 31 PVH patients (74%) underwent a secondary-stage abdominal wall reconstruction procedure to achieve a definitive abdominal wall closure. In 16 cases, a mesh enhanced procedure was used. In nine cases, the fascial edges could not be approximated, and a mesh was used as an abdominal wall substitute in inlay position. In seven cases, an anterior component separation as described by Ramirez et al. (20) was necessary to close the fascial defect. One patient required an upper thigh myocutaneous flap to reconstruct the abdominal wall (Figure 2).

One-fourth of the patients with a planned ventral hernia did not undergo secondary reconstruction for several reasons: the majority of them deemed the perioperative risk too high to attempt a procedure or the surgeon refrained from it for the same reason. Of the nine patients where a primary fascial closure was achieved, five (55%) developed a subsequent incisional hernia. Two of them underwent more than two attempts at abdominal wall reconstruction. Of the 23 patients in whom a secondary-stage reconstruction was performed, 15 (65%) eventually developed a recurrent incisional hernia. As expected, all of the patients without primary fascial closure and who did not receive a reconstruction developed a (planned ventral) hernia. In our clinical follow-up, a total of 28 (70%) patients presented with a clinically relevant abdominal hernia.

### Scarring

**Figure 3:**
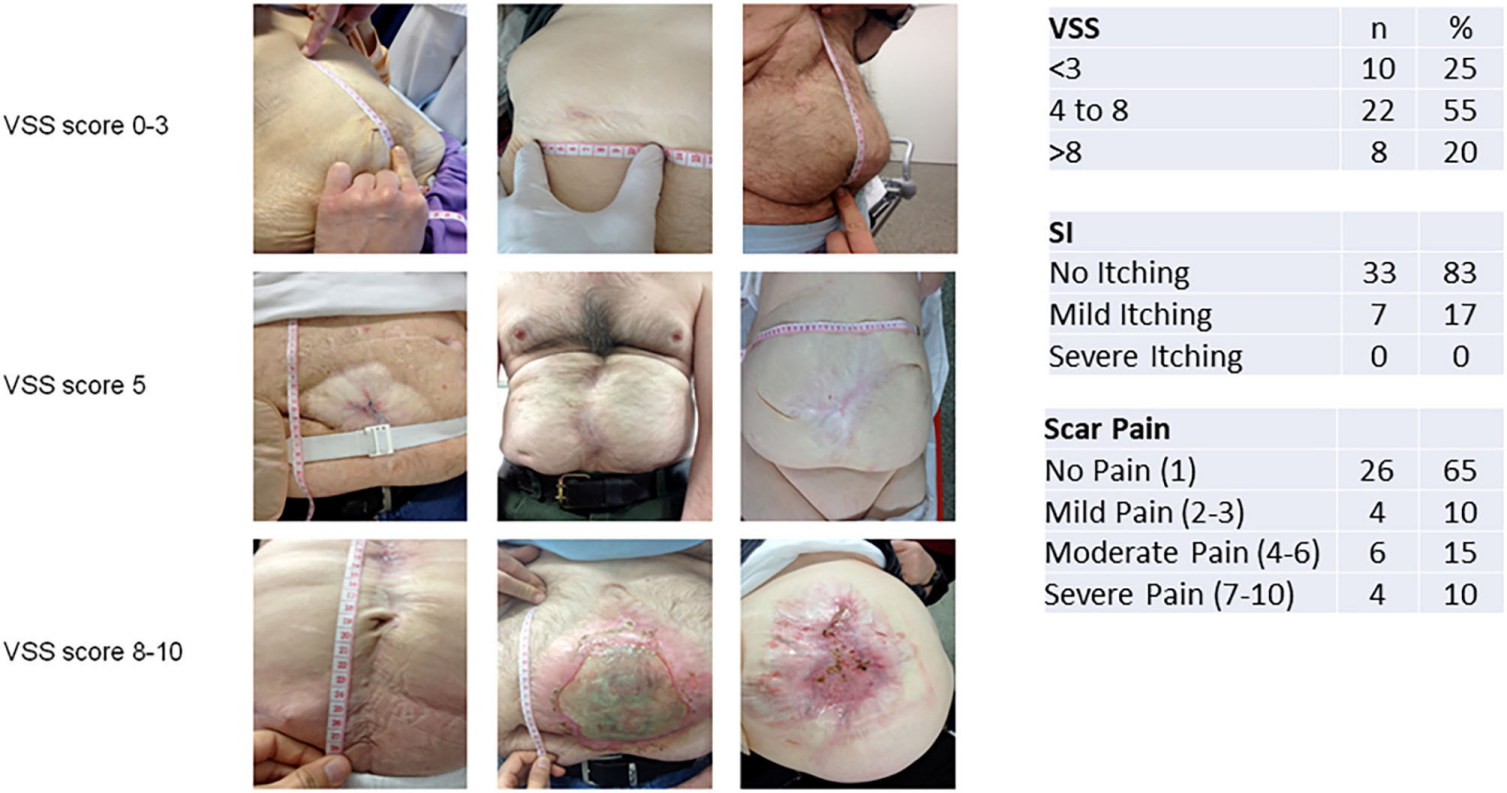
Vancouver Scar Scale (VSS) assessment combined with scar itching and pain scores. Shown are the VSS assessment scale and scar itching and scar pain results with clinical examples from our cohort.

In our cohort, 10 patients developed mild scars, with VSS < 4. Twenty-two (55%) of the patients presented with a score between 4 and 8, while eight of them had a VSS score >8 that represents significant scarring (Figure 3).

Itching was not a problem for 33 (83%) of the patients, and seven patients complained about only mild itching, none of severe itching. The majority of the patients did not report significant scar pain, with four patients reporting only mild pain (two and three on the VAS Pain Scale). Six patients complained about moderate pain (4–6 in the VAS Pain Scale), while four patients experienced severe pain with VAS > 7. Eight (20%) patients developed ulcers on the scar tissue, some of which were microbially contaminated (Figure 3, bottom picture; refer to Supplementary Material for data on bacterial contamination).

### Quality of Life

**Figure 4:**
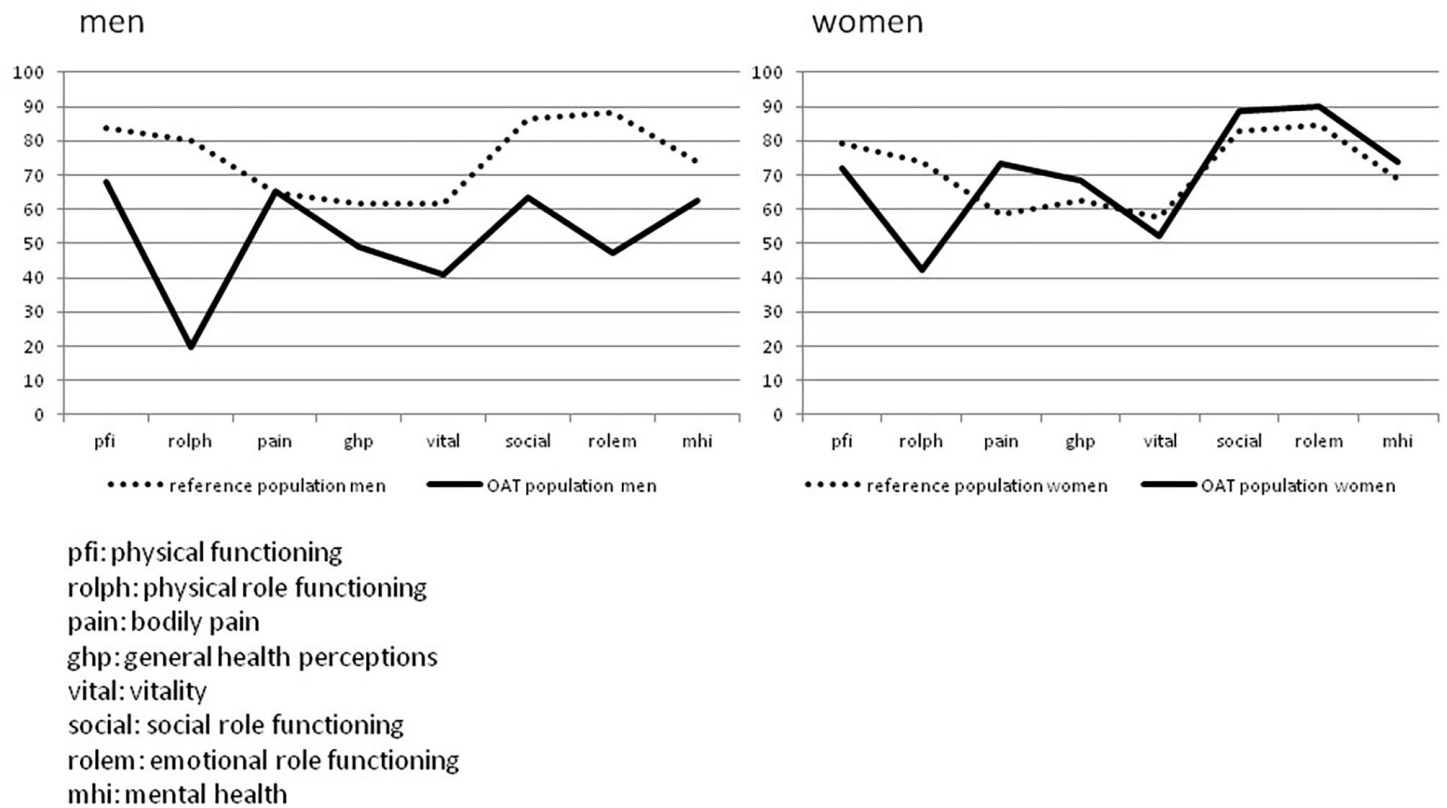
SF-36 results of open abdomen treatment (OAT) cohort (*n* = 40), visual comparison to general population. Shown are the SF-36 QoL results of men (*n* = 30) and women (*n* = 10) after OAT compared to the general population (21).

Quality of life as assessed by SF-36 showed impaired physical role functioning in men as well as in women when compared to the normal population (21). The SF-36 scores are known to be sex dependent; therefore, they are given to men and women separately (Figure 4).

It was also analyzed which clinical findings after OAT impacted on QoL as measured by the SF-36 questionnaire. The presence of a clinically evident hernia or EAF and also the factors sex, primary fascial closure, and scarring (VSS score low *vs*. high) were compared. While hernia presence, primary closure *vs*. planned ventral hernia, and scarring showed no statistically significant differences, the factors sex and especially EAF formation impacted significantly on QoL (Table 2).

**Table 2:** Analysis of clinical findings impacting on SF-36.

**Sectors SF-36**	**Sex**	**Hernia**	**PC**	**EAF**	**VSS**
	**Male vs. Female**	**Yes vs. No**	**Yes vs. No**	**Yes vs. No**	**VSS Low < 3 vs. High > 8**
Vitality	ns	ns	ns	ns	ns
Physical functioning	ns	ns	ns	ns	ns
Bodily pain	ns	ns	ns	ns	ns
General health perceptions	female^*^	ns	ns	no EAF^*^	ns
Physical role functioning	ns	ns	ns	ns	ns
Emotional role functioning	female^*^	ns	ns	no EAF^*^	ns
Social role functioning	female^*^	ns	ns	no EAF^**^	ns
Mental health	ns	ns	ns	no EAF^**^	ns
Overall physical	ns	ns	ns	ns	ns
Overall mental	female^*^	ns	ns	no EAF^*^	ns

*Table shows the comparison of mean SF-36 sector results and the following factors were compared: male vs. female sex, presence or absence of a clinically evident hernia, primary fascial closure, development of an entero-atmospheric fistula, and a low VSS score (<3) vs. a high one (>8). Non-statistically significant differences are stated as “ns.” Where a statistically significant difference was detected, it is marked accordingly, i.e., EAF formation impacts significally on the SF-36 sector “general health perceptions” [two-sided t-test*,

* p < 0.05 and

** *p < 0.01*,

*the original mean value data is given in Supplements (Supplementary Table 2)]*.

## Discussion

OAT is a specialized treatment that can prove to be life-saving for critical situations of abdominal sepsis but is inherently associated with high morbidity. Survivors of OAT face various factors that potentially limit their quality of life.

In this long-term follow-up with over 4 years after the index procedure of a single-center patient cohort after OAT, we show that several aspects of OAT must be addressed to achieve a satisfactory outcome. Overall survival was decent at best with 95/165 patients (58%). Due to the evolution of OAT at our institution which reflects the advances in OAT strategies in general, survival has improved to over 64% in the current era. The primary fascial closure rates which are the focus of surgical management (as only fascial closure as a “surgical factor” significantly reduces mortality and morbidity) were also relatively low in this historic cohort (only 9/40 patients, 23%). Recent algorithms such as the utilization of a consequent three-column approach (fascial traction, visceral protection, negative pressure wound treatment) have significantly improved primary closure rates over time not only at our institution (22). In the historic cohort reported here, a primary closure was not achieved in the majority of cases, which would not be acceptable compared to contemporary standards. Interestingly, primary fascial closure *vs*. planned ventral hernia was not a factor that impacted on quality of life. This could, in part, explain why only 75% of PVH patients were scheduled for secondary reconstruction, the reasons being mainly 2-fold: either the surgeon deemed the patient unfit for surgery or the patient refused secondary reconstruction due to lack of hernia-related complaints and/or fear of complications. More than half of the patients after successful PC and 65% of patients after a secondary reconstruction for PVH eventually developed a recurrent ventral hernia. Comparably, in the recent literature, the incidence of an incisional hernia after OAT is high, reaching up to 65% (6). This is comparable to other high-risk situations as abdominal aortic aneurysm operation (7), transplantation (23), or obesity and considerably higher than hernia incidence after elective laparotomy (5). In some of the above-mentioned instances, the use of prophylactic mesh implantation may reduce incisional hernia incidence (24). Furthermore, recent studies showed a significant reduction in incisional hernia incidence after “onlay” mesh reinforcement compared with suture only and superior to “sublay” mesh (5). As such, onlay mesh reinforcement may have the potential to improve the standard treatment for high-risk patients including OAT.

OAT may result in excessive scar tissue which may affect QoL in the long term. This is especially true for patients after OAT where PC of the fascia (and/or skin) cannot be achieved and who may be discharged with a granulating laparostomy. Excessive scarring after burn injuries is known be associated with reduced QoL and is related to disruption of daily activities, altered sleep patterns, anxiety, depression, and issues of social acceptance (15). Furthermore, hypertrophic scars might be itchy and painful and cause serious functional and cosmetic disability (25). We assessed scar formation in our follow-up cohort for the first time after OAT in a standardized manner by utilizing the Vancouver Scar Scale. Even though we found a high percentage of VSS > 3 scar formation (in 30 patients, 75%), we did not find a correlation of the VSS score (low VSS *vs*. high VSS) with QoL. This could be related to the fact that no patient experienced severe scar itching and only 10% experienced severe scar pain, which are factors known to impair QoL after burn injuries and have since been added to the VSS score (26). Although previous studies did show a correlation between VSS and pain as well as itching, we could not detect such correlation. However, pain and itching correlated significantly among themselves, with Pearson's *P* = 0.533. While the subject of scar tissue development after OAT is much less well-understood as scarring after burn damage, further research in this area may provide ways of minimizing scar-related problems, ensuring better aesthetic results as well as less scar tissue complications in these patients. One finding that the authors noticed was a colonization of multi-resistant bacterial strains in unstable scars of some patients (especially after granulating laparostomy; see Figure 1, bottom picture, and Supplementary Figure 3), which have to be addressed before secondary reconstruction is attempted. Concerning quality of life, we found that the sector “physical role functioning” was most impaired after OAT—especially in men—compared to the general population. This is not a surprising finding in our cohort because men, in particular, may find persisting disabilities after OAT a hindrance to former job-related physical labor or activities in daily life. The inherent sex difference in QoL, when assessed with SF-36, was also seen in our data with better QoL reported by 10 females of the cohort. The biggest negative impact on QoL was seen in patients with EAF formation; here the sectors “general health perception,” “emotional and social role functioning,” and “mental health” as well as the overall mental status score were negatively affected. We conclude that the avoidance of EAF formation, best achieved by the consequent use of visceral protection and early midline closure, is paramount not only for survival and morbidity but also to preserve QoL in OAT patients. Naturally, our study has several limitations: the small sample size and single-center setup limit generalizability in some aspects. It could be argued that a selection bias may have distorted the clinical follow-up because some patients were reached but did not want to participate in the clinical follow-up study. According to the patients' statements, several reasons were mentioned: some patients avoided hospitalization due to the previous traumatic experience, some argued that the distance to our center was too far, and several patients did not want a follow-up due to lack of complaints. It is therefore conceivable that especially the last group manages well with a stable abdomen and that the rate of patients with planned ventral hernia may be distortedly high in our follow-up cohort. The small sample size may have impacted on the SF-36 analysis especially in the subgroups and hindered the detection of all potential factors that logically would influence QoL (such as PC of the fascia). For the same reason, multivariate analysis was not feasible in this cohort, which would be interesting in a larger, multicentric database ^1^.

To summarize, we show that an early-era approach to OAT before the implementation of a structured concept such as the “Koblenz algorithm” with (a) fascial traction, (b) visceral protection, and (c) negative pressure therapy resulted in relevant mortality and morbidity. Our follow-up strategy identified a significant proportion of patients that would qualify for a secondary reconstructive procedure, but only about 2/3 of patients discharged with a planned ventral hernia wanted reconstructive surgery and were deemed fit. Of all clinical factors tested, only sex and EAF formation impacted on quality of life, which was generally lower in OAT patients compared to the general population concerning bodily role functioning. Hernia rates after PC were still high with over 50%, and prophylactic measures such as a prophylactic mesh implantation upon fascial closure should be discussed in the future analogous to other major and emergency abdominal procedures.

## Data Availability Statement

The original contributions generated for this study are included in the article/Supplementary Material, further inquiries can be directed to the corresponding author/s.

## Ethics Statement

The studies involving human participants were reviewed and approved by Ethikkomitee der Medizinischen Fakultät Bonn. The patients/participants provided their written informed consent to participate in this study.

## Author Contributions

MW and SM contributed to the conception and design of the study. AT and AJ performed most of the data collection. MW and AT drafted the manuscript. All the authors contributed to data collection and manuscript revision and approved the manuscript.

## Conflict of Interest

The authors declare that the research was conducted in the absence of any commercial or financial relationships that could be construed as a potential conflict of interest.

## Footnotes

^1^Available online at: https://www.eurahs.eu/.

## Abbreviations

OAT: open abdomen treatment
QoL: quality of life
EAF: entero-atmospheric fistula
PC: primary fascial closure
PVH: planned ventral hernia
VSS: Vancouver Scar Score.
