# Supplementary material for: Long Term Outcome After Open Abdomen Treatment: Function and Quality of Life
Source: Front Surg. 2021 Mar 29;8:590245. doi: 10.3389/fsurg.2021.590245 (PMC8039509; doi:10.3389/fsurg.2021.590245)
Supplement: Supplementary file 1 [file Data_Sheet_1.DOCX]

**Figure S1:** Original patient cohort, survival flow chart and follow up study participation

**Figure S1** depicts a flow chart of the original patient cohort after OAT of 165 patients with 95 survivors, 40 of which participated in the clinical follow up study.

**Table S2**: QoL analysis (Mean value comparisons of SF-36 sectors related to clinical factors)

|  | Sex | | IndOAT | | PrimClos | | EAF | | Hernia_clin | | VSS lo/high | |
| --- | --- | --- | --- | --- | --- | --- | --- | --- | --- | --- | --- | --- |
|  | female | male | Peritonitis | other | no PC | PC | no EAF | EAF | no Hernia | Hernia | VSSlo=<3 | VSShigh=>3 |
|  | Mean | Mean | Mean | Mean | Mean | Mean | Mean | Mean | Mean | Mean | Mean | Mean |
| SF-36 Physical functioning. (0-100) | 72,17 | 67,76 | 69,90 | 64,29 | 64,89 | 84,38 | 69,74 | 65,00 | 77,27 | 65,60 | 75,21 | 66,17 |
| SF-36 Physical role functioning (0-100) | 42,50 | 19,83 | 28,13 | 14,29 | 22,58 | 37,50 | 29,69 | 7,14 | 27,27 | 25,00 | 15,63 | 27,50 |
| SF-36 Bodily pain (0-100) | 73,30 | 65,21 | 68,94 | 59,71 | 68,45 | 62,75 | 71,13 | 49,71 | 53,73 | 72,61 | 61,25 | 67,80 |
| SF-36 General health perceptions (0-100) | 68,60 | 49,00 | 53,97 | 54,29 | 54,35 | 52,75 | 57,78 | 36,86 | 56,73 | 52,96 | 55,63 | 52,33 |
| SF-36 Vitality (0-100) | 52,00 | 41,03 | 44,38 | 41,43 | 45,00 | 39,38 | 46,41 | 32,14 | 39,55 | 45,54 | 45,00 | 43,17 |
| SF-36 Social role functioning (0-100) | 88,75 | 63,36 | 68,75 | 75,00 | 70,16 | 68,75 | 76,17 | 41,07 | 65,91 | 71,43 | 64,06 | 70,42 |
| SF-36 Emotional role functioning (0-100) | 90,00 | 47,13 | 58,33 | 57,14 | 62,37 | 41,67 | 66,67 | 19,05 | 57,58 | 58,33 | 41,67 | 61,11 |
| SF-36 Mental Health(0-100) | 74,00 | 62,76 | 65,13 | 68,00 | 65,55 | 66,00 | 70,38 | 44,00 | 60,00 | 67,86 | 60,50 | 67,07 |
| Physical Summary Score | 42,40 | 39,52 | 40,98 | 36,96 | 39,18 | 44,45 | 40,76 | 37,98 | 41,16 | 39,90 | 41,40 | 39,45 |
| Mental Summary Score | 53,29 | 42,09 | 44,52 | 47,00 | 46,12 | 40,47 | 47,99 | 31,14 | 42,04 | 46,11 | 40,81 | 45,93 |

**Table S2** gives all mean value results of the SF-36 sectors, analyzed in relation to the clinical factors sex, indication for OAT, primary closure, enteroatmospheric fistula, hernia incidence and VSS score.

**Figure S3:** Bacterial colonization of abdominal scar after OAT in patients with VSS score >8

**Figure S3** shows the results of microbial analysis of samples derived from the wounds/abdominal scars of the 11 patients who displayed a VSS>8 (severe scarring). Of note, 27% showed colonization with multi-resistant strains such as multi-resistant staphylococcus strains (MRSA).
